# Supplementary material for: Regulation of cadherin dimerization by chemical fragments as a trigger to inhibit cell adhesion
Source: Commun Biol. 2021 Sep 7;4:1041. doi: 10.1038/s42003-021-02575-3 (PMC8423723; doi:10.1038/s42003-021-02575-3)
Supplement: Supplementary file 3 — Description of Supplementary Files [file 42003_2021_2575_MOESM3_ESM.pdf]

Description of Additional Supplementary Files

File name: Supplementary Data 1

Description: This file contains all source data for main figures. The excel file has several sheets that reflect the figure numbers.
